# Neurochemical and Behavior Deficits in Rats with Iron and Rotenone Co-treatment: Role of Redox Imbalance and Neuroprotection by Biochanin A

**DOI:** 10.3389/fnins.2017.00657

**Published:** 2017-11-23

**Authors:** Lijia Yu, Xijin Wang, Hanqing Chen, Zhiqiang Yan, Meihua Wang, Yunhong Li

**Affiliations:** ^1^Department of Neurology, Xinhua Hospital, Shanghai Jiao Tong University School of Medicine, Shanghai, China; ^2^School of Biotechnology and Food Engineering, Hefei University of Technology, Hefei, China; ^3^Shanghai Laboratory Animal Center, Chinese Academy of Sciences, Shanghai, China

**Keywords:** parkinson's disease, iron, roteone, redox balance, biochanin A

## Abstract

Increasing evidences show that the etiology of Parkinson's disease (PD) is multifactorial. Studying the combined effect of several factors is becoming a hot topic in PD research. On one hand, iron is one of the essential trace metals for human body; on the other hand, iron may be involved in the etiopathogenesis of PD. In our present study, the rats with increased neonatal iron (120 μg/g bodyweight) supplementation were treated with rotenone (0.5 mg/kg) when they were aged to 14 weeks. We observed that iron and rotenone co-treatment induced significant behavior deficits (time-dependent) and striatal dopamine depletion in the male and female rats, while they did not do so when they were used alone. No significant change in striatal 5-hydroxytryptamine content was observed in the male and female rats with iron and rotenone co-treatment. Also, iron and rotenone co-treatment significantly decreased substantia nigra TH expression in the male rats. Furthermore, co-treatment with iron and rotenone significantly induced malondialdehyde increase and glutathione decrease in the substantia nigra of male and female rats. There was no significant change in cerebellar malondialdehyde and glutathione content of the rats co-treated with iron and rotenone. Interestingly, biochanin A significantly attenuated striatal dopamine depletion and improved behavior deficits (dose-dependently) in the male and female rats with iron and rotenone co-treatment. Biochanin A treatment also significantly alleviated substantia nigra TH expression reduction in the male rats co-treated with iron and rotenone. Finally, biochanin A significantly decreased malondialdehyde content and increased glutathione content in the substantia nigra of male and female rats with iron and rotenone co-treatment. Our results indicate that iron and rotenone co-treatment may result in aggravated neurochemical and behavior deficits through inducing redox imbalance and increased neonatal iron supplementation may participate in the etiopathogenesis of PD. Moreover, biochanin A may exert dopaminergic neuroprotection by maintaining redox balance.

## Introduction

Parkinson's disease (PD) is a common neurodegenerative disorder clinically characterized by four major hallmarks including resting tremor, rigidity, bradykinesia and postural instability (Lang and Lozano, 1998; Dauer and Przedborski, 2003; Fiesel et al., 2015). Accumulating studies have showed that the etiology of Parkinson's disease (PD) is multifactorial (Kidd, 2000; Connolly and Lang, 2014; Mitsuyama et al., 2015; Richter et al., 2017). Environmental factors are shown to play an important role in the etiopathogenesis of PD (Olanow and Tatton, 1999; Tanner et al., 1999; Ascherio and Schwarzschild, 2016). In recent years, studying the combined effect of several risk factors is being become a hot topic in PD research.

Iron is one of the essential trace metals for human body especially for neural development. Iron deficiency negatively impacts on myelinogenesis, synthesis of neurotransmitters and construction of neural connections (Beard, 2003; Stankiewicz et al., 2007). Insufficient iron supplement also results in a number of neurological and psychiatric conditions such as pediatric restless leg syndrome and attention deficit hyperactivity disorder (Millichap, 2008; Benton, 2010). In addition, anemia due to iron deficiency in infants contributes to cognitive and social impairments (Carter et al., 2010; Wang L. et al., 2016). Because of the essential role of iron in neurological as well as overall development, it has been recommended that breast milk which contains iron nutrition is preferred benefit for infants and may be replaced with iron-fortified formula to those who cannot receive breastfeed. However, studies have shown that increased neonatal iron supplementation could result in increased iron content in the substantia nigra and subsequent nigrostriatal dopaminergic neurodegeneration in aging rats (Kaur et al., 2007; Chen H. et al., 2015). Rotenone, a mitochondrial complex I inhibitor, has been extensively used as pesticides in a rural environment. Rotenone is being widely employed into PD models because of its highly selective toxicity on dopaminergic neurons and effective reproduction of pathological and clinical features of PD (Betarbet et al., 2000; Sherer et al., 2003b; Cannon et al., 2009; Sanders and Greenamyre, 2013; Jagmag et al., 2016). However, little is known about whether increased neonatal iron supplementation enhances susceptibility of dopaminergic neurons to subsequent exposure of rotenone.

In our present study, the rats with increased neonatal iron (120 μg/g bodyweight) supplementation were treated with rotenone (0.5 mg/kg) for 35 days when they were aged to 14 weeks. We investigated the combined effect of iron and rotenone treatment and mechanism of action on behavioral and neurochemical indexes in male and female rats. Biochanin A, an O-methylated isoflavone, is classified as phytoestrogen due to its similar chemical structure with mammalian estrogen. Moreover, biochanin A has been suggested to be protective in several *in vitro* and *in vivo* models (Chen et al., 2007; Su et al., 2013; Wang J. et al., 2016). Therefore, in this study, we also investigated biochanin A's effect and mechanism of action in male and female rats co-treated with iron and rotenone.

## Materials and methods

### Animals and treatment

All animals were from Sino-British SIPPR/BK Lab Animal LTD (Shanghai, People's Republic of China). Sprague-Dawley rat pups were fed either saline vehicle or carbonyl iron daily by oral gavage from days 10 to 17 post-partum. Based on the previous studies (Kaur et al., 2007; Stankiewicz et al., 2007; Chen H. et al., 2015), the rat pups were fed with increased iron (120 μg/g bodyweight). The rats were aged to 14 weeks. Then, rotenone, emulsified in sunflower oil at 0.5 mg/mL, was given intraperitoneally, at 1 mL/kg once a day for 35 days, to the rats (Wang et al., 2015). Biochanin A was dissolved in dimethyl sulfoxide (DMSO) and administered intraperitoneally (0.1 ml/100 g/day) to different groups of rats at two different concentrations of 3 and 30 mg/kg. Together with rotenone injection, the rats were continuously treated with biochanin A for 35 days. To investigate the combined effect of iron and rotenone treatment and mechanism of action on behavioral and neurochemical indexes in male and female rats, rats (male and female) were randomly divided into four groups: Veh group (rats co-treated with saline and sunflower oil), Ir group (rats co-treated with iron and sunflower oil), Rot group (rats co-treated with saline and rotenone), and Ir+Rot group (rats co-treated with iron and rotenone). To investigate biochanin A's effect and mechanism of action in male and female rats co-treated with iron and rotenone, rats (male and female) were randomly divided into five groups: Veh group (rats co-treated with saline, sunflower oil and DMSO), Ir+Rot group (rats co-treated with iron, rotenone and DMSO), Ir+Rot+BA3 group [rats co-treated with iron, rotenone and biochanin A (3 mg/kg)], Ir+Rot+BA30 group [rats co-treated with iron, rotenone and biochanin A (30 mg/kg)], and BA30 group [rats co-treated with saline, sunflower oil and biochanin A (30 mg/kg)]. For research involving biohazards, biological select agents, toxins (including rotenone), restricted materials or reagents, the standard biosecurity or institutional safety procedures were carried out in our experiments. The use of toxic or biohazards substances was approved by the Health, Safety and environment (HSE) Committee of Xinhua Hospital Affiliated to Shanghai Jiao Tong University School of Medicine. This study was reviewed and approved by the Ethical Committee of Xinhua Hospital Affiliated to Shanghai Jiao Tong University School of Medicine. All experiments were carried out in accordance with the approved guidelines and regulations of the National Institutes of Health for the care and use of laboratory animals. All attempts were made to minimize the number of animals used and their suffering.

### Behavior tests

Rotarod and open-field tests were performed to evaluate rat behavior on the 15th and 45th day after the last injection of rotenone. The rotarod apparatus required a roller, a power source to turn the roller and four separators that divided the roller into equal-sized compartments. Rats were placed onto the rotating rod and trained at accelerated speeds of 5, 10, and 15 rotations per minute (rpm). After completing the training, each rat was given three trials at each rotarod speed and the latency time to fall was recorded. To assess the locomotor activity, the rats were tested in an open field chamber (100 × 100 × 50 cm^3^) with the floor divided into 25 equal squares of 20 × 20 cm^2^, which is made of wood covered with impermeable formica. Each rat was initially placed in the center of the open field to acclimatize for 10 min and then behavioral parameters including crossing number (entering of another square with all four paws) and rearing number (rearing with and without wall contact namely standing only on hind legs) were measured during a period of 30 min.

### Neurochemical analysis

High-performance liquid chromatography (HPLC) with electrochemical detection (ECD) (HPLC-ECD) was used to the biochemical analysis of neurotransmitters in the rat striata as previously described (McNaught et al., 2004). Briefly, rat striata were quickly removed on ice and weighed. Striata were homogenized (10% wt/vol) in ice-cold homogenization buffer containing perchloric acid (0.1 mol/L) using sonication and 3,4-dihydroxybenzylamine is applied to be an internal control. After lysis, samples were centrifuged at 4°C for 10 min and the collected supernatants were then assayed for dopamine and 5-hydroxytryptamine content through HPLC-ECD equipped with a column of 5 μm spherical C18 particles. The mobile phase consisted of 4.5% acetonitrile, 0.1 M phosphate buffer (pH 2.6) containing 2.5%methanol and 0.2 mM octane sulfonic acid. The content of each neurotransmitter was expressed as ng/g equivalent striatal tissue.

### Western blotting

The substantia nigra tissues of rats were separated and homogenized in cold lysis buffer. Protein lysates were adjusted to equal protein concentrations using bicinchoninic acid (BCA) protein assay kit (Beyotime Institute of Biotechnology, Shanghai, China). After being separated by 10% sodium dodecyl sulfate-polyacrylamide gels (SDS-PAGE), protein samples were transferred onto polyvinylidene fluoride membranes (Millipore, Bedford, MA, USA). Then, the membranes were blocked with blocking solution for 1 h and incubated with primary rabbit anti-tyrosine hydroxylase (TH) antibody (Abcam, Cambridge, UK) or rabbit anti-β-Actin antibody (Abcam) at 4°C overnight. Subsequently, the membranes were washed and incubated with horseradish peroxidase (HRP)-conjugated secondary antibodies for another 1 h at room temperature. Finally, detection of protein bands was performed by using an enhanced chemiluminescence (ECL) assay kit (EMD Millipore, Billerica, MA, USA). The density of bands was quantified by using ImageJ software (National Institutes of Health, USA) and results presented the ratio of density of the target protein to β-actin as densitometric relative units.

### Determination of malondialdehyde and glutathione levels

The levels of malondialdehyde and glutathione were measured by commercially available kits (Cayman Chemical Co., Ann Arbor, MI, USA) according to the manufacturer's instructions. Substantia nigra tissues were quickly removed on ice and homogenized (10% wt/vol) with radioimmunoprecipitation assay (RIPA) homogenizing buffer containing a protease inhibitor for protein extraction. Then samples were centrifuged at 1,600 g for 10 min at 4°C and supernatants were collected. For malondialdehyde assay, thiobarbituric acid (TBA) reacts with malondialdehyde to generate a thiobarbituric acid reactive substance which can be quantified. Samples or standards (100 μl) were added to trichloroacetic acid and thiobarbituric acid reactive substances reagent and then the mixture solutions were boiled for 1 h. After incubating on the ice for 10 min to stop reaction, samples were centrifuged and the absorbance values of the supernatants were read at 540 nm. For glutathione assay, the determination was based on the enzymatic recycling method. Since glutathione reductase is used in the Cayman GSH assay, both GSH and GSSG are measured and the assay reflects total glutathione. Briefly, 100 μl of supernatant from substantia nigra sample was deproteinated with 100 μl metaphosphoric acid reagent, added by triethanolamine reagent (50 μl/ml, 4 M) and pipetted 50 μl of the solution. Then, this was followed by the addition of 150 μl of freshly prepared Assay Cocktail consisting of 11.25 ml of MES Buffer, 2.1 ml of reconstituted Enzyme Mixture, 0.45 ml of reconstituted Cofactor Mixture, 0.45 ml of reconstituted DTNB [5,5′-dithio-bis-(2-nitrobenzoic acid)] and 2.3 ml of water and incubated for 25 min. The absorbance value was read at 405 nm.

### Statistical analysis

Data were expressed as the mean ± standard error of the mean (SEM). Results were analyzed by two-tailed Student's *t*-test for comparison between two groups and an analysis of variance (ANOVA) followed by Bonferroni *post hoc* test for comparison between more than two groups. Normality of sample distribution and homogeneity of variances were tested before each ANOVA. A value of *p* < 0.05 was considered to be statistically significant.

## Results

### Effect of iron and rotenone co-treatment on behavioral and neurochemical indexes of male and female rats

**Figure 1:**
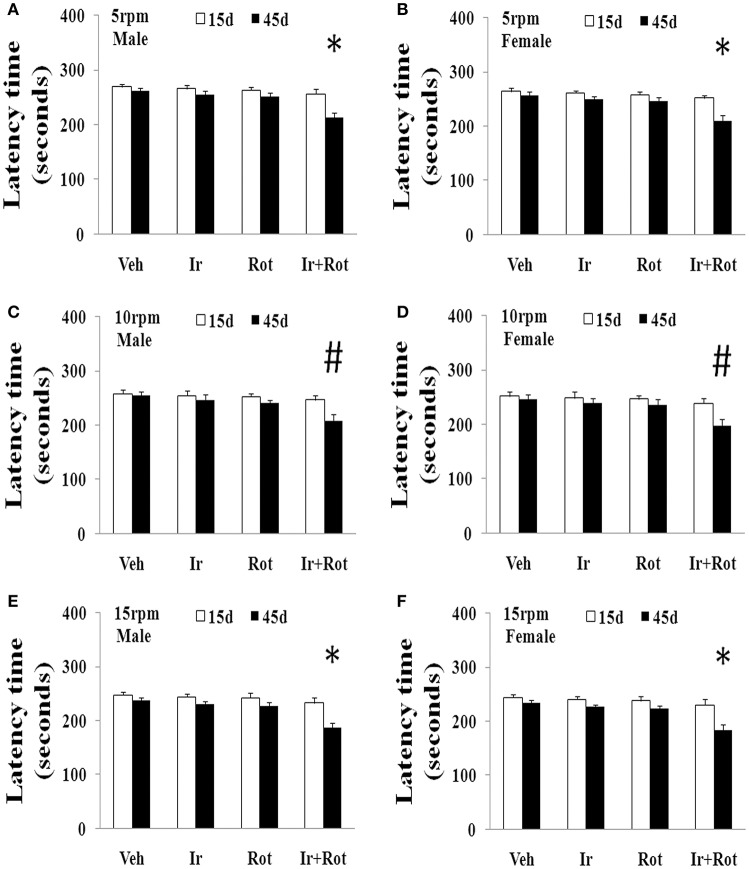
Effect of iron and rotenone co-treatment on motor behavior of male **(A,C,E)** and female **(B,D,F)** rats in rotarod test (**A,B**: 5 rpm; **C,D**: 10 rpm; **E,F**: 15 rpm). Results are expressed as mean ± SEM. *N* = 9. Latency time was analyzed using multi-factor ANOVA followed by Bonferroni *post hoc* test. ^#^*p* < 0.05, compared with the rats treated with vehicle, iron or rotenone; ^*^p < 0.01, compared with the rats treated with vehicle, iron or rotenone. Veh, vehicle; Ir, iron; Rot, rotenone.

**Figure 2:**
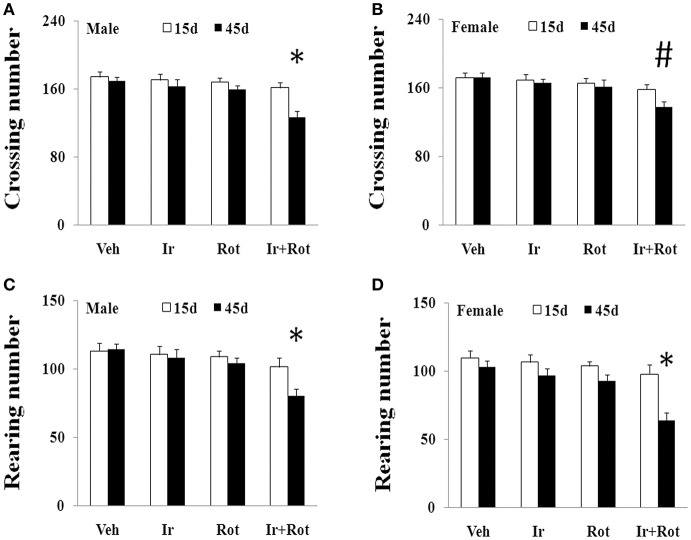
Effect of iron and rotenone co-treatment on motor behavior of male **(A,C)** and female **(B,D)** rats in open field test (**A,B**: crossing number; **C,D**: rearing number). Results are expressed as mean ± SEM. *N* = 9. Crossing and rearing number were analyzed using multi-factor ANOVA followed by Bonferroni *post hoc* test. ^#^*p* < 0.05, compared with the rats treated with vehicle, iron or rotenone; ^*^p < 0.01, compared with the rats treated with vehicle, iron or rotenone. Veh, vehicle; Ir, iron; Rot, rotenone.

**Figure 3:**
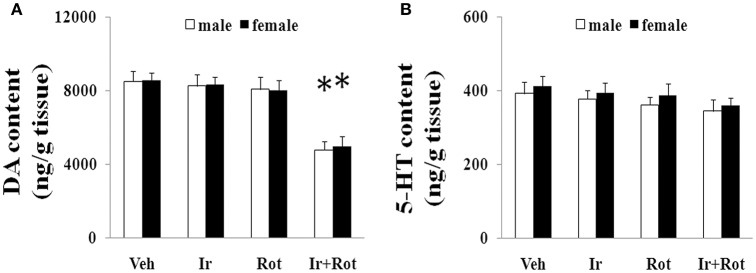
Effect of iron and rotenone co-treatment on striatal dopamine **(A)** and 5-hydroxytryptamine **(B)** conent in male and female rats. Results are expressed as mean ± SEM. *N* = 9. DA and 5-HT content were analyzed using multi-factor ANOVA followed by Bonferroni *post hoc* test. ^*^p < 0.01, compared with the rats treated with vehicle, iron or rotenone. Veh, vehicle; Ir, iron; Rot, rotenone. DA, dopamine; 5-HT, 5-hydroxytryptamine.

**Figure 4:**
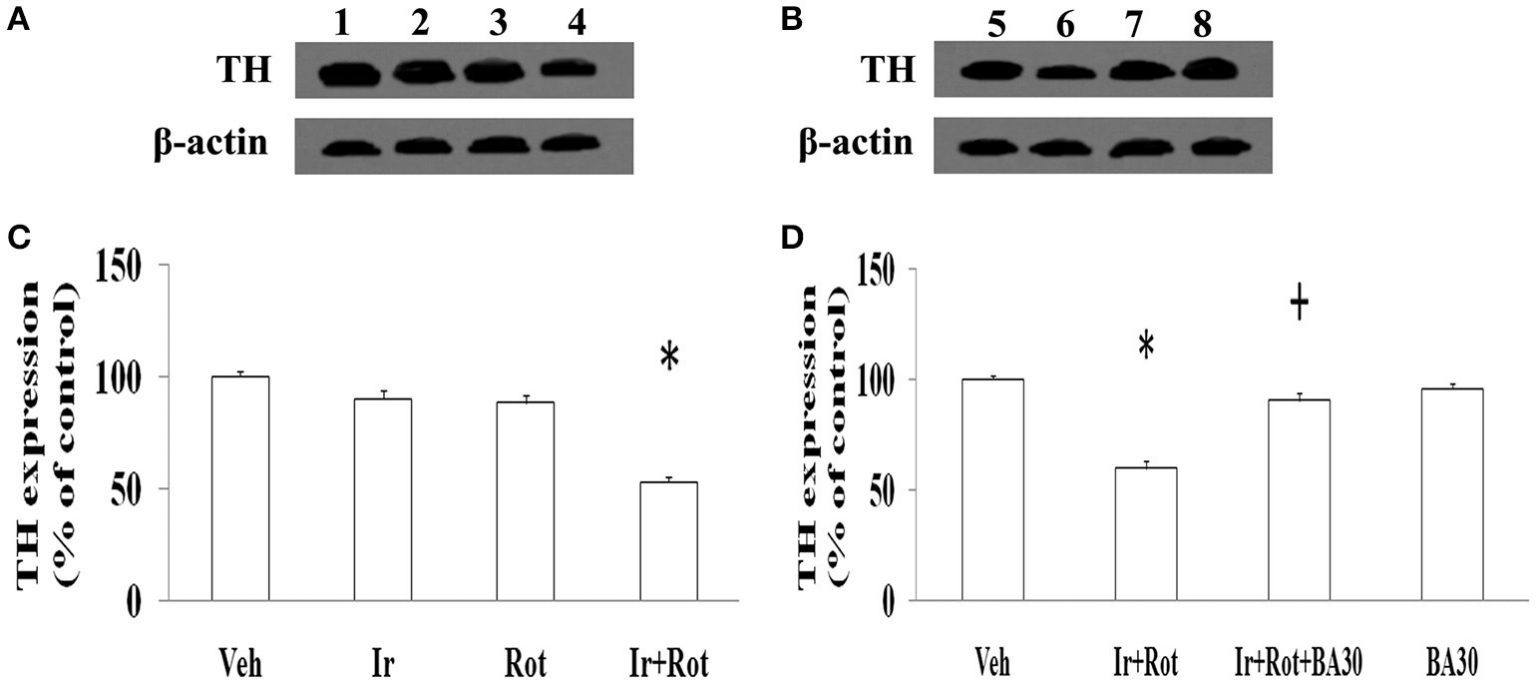
Effect of iron, rotenone and biochanin A co-treatment on substantia nigra TH expression in male rats. Results are expressed as mean ± SEM. *N* = 9. TH expression was analyzed using multi-factor ANOVA followed by Bonferroni *post hoc* test. ^*^p < 0.01**(C)**, compared with the rats treated with vehicle, iron or rotenone. ^*^p < 0.01**(D)**, compared with the rats treated with vehicle. ^+^*p* < 0.01, compared with the rats co-treated with iron and rotenone. 1, Veh1(saline)+Veh2(sunflower oil); 2, Ir+Veh2; 3, Veh1+Rot; 4, Ir+Rot; 5, Veh1+Veh2+Veh3(DMSO); 6, Ir+Rot+Veh3; 7, Ir+Rot+BA30; 8, Veh1+Veh2+BA30; Veh, vehicle; Ir, iron; Rot, rotenone; BA30, biochanin A (30 mg/kg).

To evaluate the effect of iron and rotenone co-treatment on motor behavior in male and female rats, the rotarod and open field tests were performed on rats on the 15th and 45th day after the last injection of rotenone. As shown in Figures 1, 2, no significant behavior change was observed in the male and female rats treated with iron (or rotenone) alone compared with the vehicle-treated rats on both the 15th and the 45th day after rotenone injection. However, iron and rotenone co-treatment significantly decreased the latency time [male and female: *p* < 0.01 (5 and 15 rpm); *p* < 0.05(10 rpm)] in rotarod test and the number of crossing and rearing [male: *p* < 0.01 (crossing and rearing); female: *p* < 0.05 (crossing), *p* < 0.01 (rearing)] in open field test in the male and female rats on the 45th day but did not significantly decrease them on the 15th day compared with the rats treated with vehicle, iron or rotenone (Figures 1, 2). Next, we investigated the effect of iron and rotenone co-treatment on striatal neurotransmitters of male and female rats. In accordance with behavior tests, no significant striatal dopamine depletion was observed in the male and female rats treated with iron (or rotenone) alone in comparison with the vehicle-treated rats (Figure 3A). However, iron and rotenone co-treatment significantly decreased striatal dopamine content in the male and female rats compared with the rats treated with vehicle, iron or rotenone (Figure 3A) (*p* < 0.01). Even though male and female rats were co-treated with iron and rotenone, no significant change in striatal 5-hydroxytryptamine level was observed in the rats compared with those treated with vehicle, iron or rotenone (Figure 3B). In addition, iron and rotenone co-treatment significantly decreased substantia nigra TH expression in the male rats compared with the rats treated with vehicle, iron, or rotenone (*p* < 0.01, Figures 4A,C). No significant change was observed in the substantia nigra TH expression of male rats treated with iron (or rotenone) alone in comparison with the vehicle-treated rats (Figures 4A,C).

### Effect of iron and rotenone co-treatment on substantia nigra malondialdehyde and glutathione content in male and female rats

**Figure 5:**
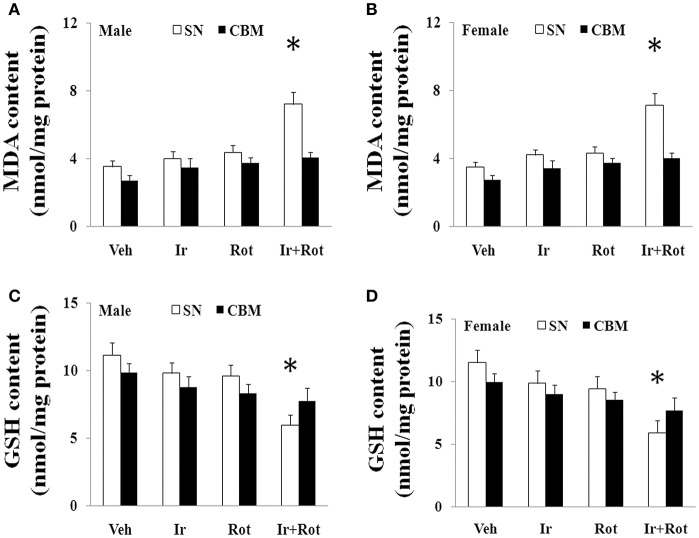
Effect of iron and rotenone co-treatment on malondialdehyde **(A,B)** and glutathione **(C,D)** conent in the substantia nigra and cerebellum of male **(A,C)** and female **(B,D)** rats. Results are expressed as mean ± SEM. *N* = 9. MDA and GSH content were analyzed using multi-factor ANOVA followed by Bonferroni *post hoc* test. ^*^p < 0.01, compared with the rats treated with vehicle, iron or rotenone. Veh, vehicle; Ir, iron; Rot, rotenone. MDA, malondialdehyde; GSH, glutathione; SN, substantia nigra; CBM, cerebellum.

Oxidative stress has been shown to play an important role in iron (or rotenone)-induced neurodegeneration (Betarbet et al., 2000; Stankiewicz et al., 2007), so we measured the content of malondialdehyde and glutathione in the substantia nigra of male and female rat to investigate the potential mechanism underlying behavioral and neurochemical deficits induced by iron and rotenone co-treatment. As shown in Figure 5, no significant change in the content of malondialdehyde and glutathione was observed in the substantia nigra of male and female rats treated with iron (or rotenone) alone in comparison with the vehicle-treated rats. However, iron and rotenone co-treatment significantly increased malondialdehyde content (*p* < 0.01) and decreased glutathione content (*p* < 0.01) in the substantia nigra of male and female rats compared with the rats treated with vehicle, iron or rotenone (Figure 5). In addition, even though male and female rats were co-treated with iron and rotenone, no significant change in malondialdehyde and glutathione content was observed in the cerebellum of rats compared with the rats treated with vehicle, iron or rotenone (Figure 5).

### Effect of biochanin a on behavioral and neurochemical indexes in male and female rats with iron and rotenone co-treatment

**Figure 6:**
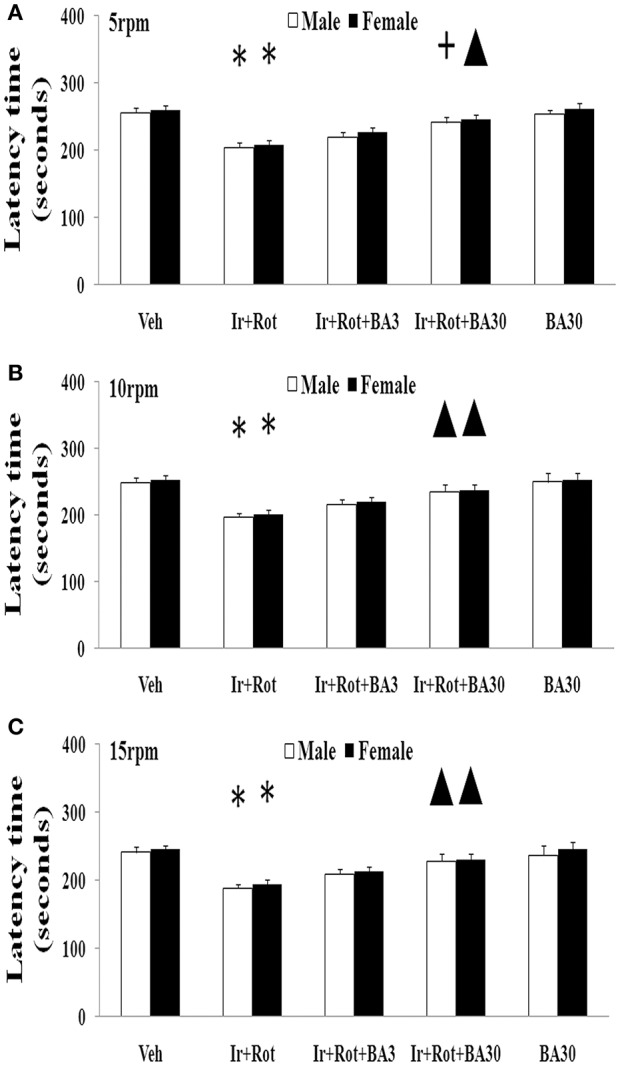
Effect of biochanin A on motor behavior (rotarod test) in male and female rats with iron and rotenone co-treatment (**A**: 5 rpm; **B**: 10 rpm; **C**: 15 rpm). Results are expressed as mean ± SEM. *N* = 9. Latency time was analyzed using multi-factor ANOVA followed by Bonferroni *post hoc* test. ^*^p < 0.01 compared with the vehicle-treated rats. ^▲^*p* < 0.05, compared with the rats co-treated with iron and rotenone. ^+^*p* < 0.01, compared with the rats co-treated with iron and rotenone. Veh, vehicle; Ir, iron; Rot, rotenone; BA3, biochanin A (3 mg/kg); BA30, biochanin A (30 mg/kg).

**Figure 7:**
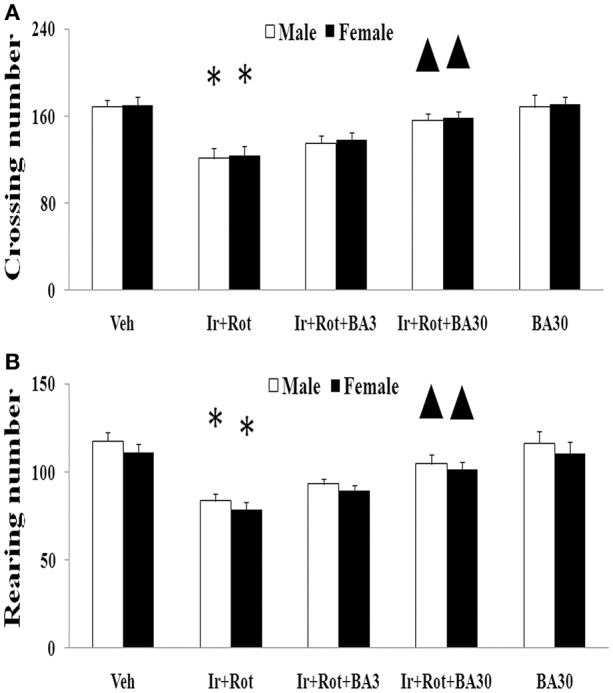
Effect of biochanin A on motor behavior (open field test) in male and female rats with iron and rotenone co-treatment (**A**: crossing number; **B**: rearing number). Results are expressed as mean ± SEM. *N* = 9. Crossing and rearing number were analyzed using multi-factor ANOVA followed by Bonferroni *post hoc* test. ^*^p < 0.01 compared with the vehicle-treated rats. ^▲^*p* < 0.05 compared with the rats co-treated with iron and rotenone. Veh, vehicle; Ir, iron; Rot, rotenone; BA3, biochanin A (3 mg/kg); BA30, biochanin A (30 mg/kg).

**Figure 8:**
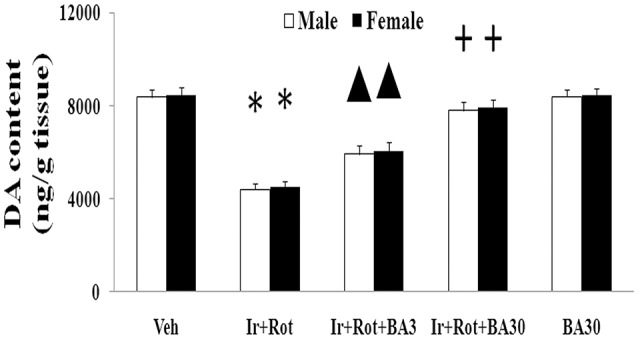
Effect of biochanin A on striatal dopamine conent in male and female rats with iron and rotenone co-treatment. Results are expressed as mean ± SEM. *N* = 9. DA content was analyzed using multi-factor ANOVA followed by Bonferroni *post hoc* test. ^*^p < 0.01, compared with the vehicle-treated rats. ^▲^*p* < 0.05, compared with the rats co-treated with iron and rotenone. ^+^*p* < 0.01, compared with the rats co-treated with iron and rotenone. DA, dopamine; Veh, vehicle; Ir, iron; Rot, rotenone; BA3, biochanin A (3 mg/kg); BA30, biochanin A (30 mg/kg).

Biochanin A has been suggested to be protective in several *in vitro* and *in vivo* models (Chen et al., 2007; Su et al., 2013; Wang J. et al., 2016). Therefore, we further investigated the effect of biochanin A in the rats co-treated with iron and rotenone. As shown in Figures 6, 7, biochanin A (30 mg/kg) administration significantly alleviated the reduction of latency time (male: 5 rpm: *p* < 0.01, 10, and 15 rpm: *p* < 0.05; female: *p* < 0.05) and crossing and rearing number (*p* < 0.05) in the male and female rats co-treated with iron and rotenone, although no significant behavior change but a trend toward improvement was observed in the rats co-treated with iron and rotenone after biochanin A (3 mg/kg) administration compared with the vehicle-treated rats. In accordance with behavior tests, biochanin A treatment significantly alleviated striatal dopamine depletion [*p* < 0.05 (3 mg/kg), *p* < 0.01 (30 mg/kg)] in the male and female rats co-treated with iron and rotenone in comparison with the Ir+Rot group (Figure 8). In addition, there was significant difference (*p* < 0.01) in striatal dopamine content between the Ir+Rot+BA3 and Ir+Rot+BA30 group, indicating that biochanin A's effect on striatal dopamine content was dose-dependent in the male and female rats co-treated with iron and rotenone. Furthermore, biochanin A (30 mg/kg) treatment significantly alleviated substantia nigra TH expression reduction (*p* < 0.01) in the male rats co-treated with iron and rotenone compared with the Ir+Rot group (Figures 4B,D).

### Effect of biochanin a on substantia nigra malondialdehyde and glutathione conent in male and female rats with iron and rotenone co-treatment

**Figure 9:**
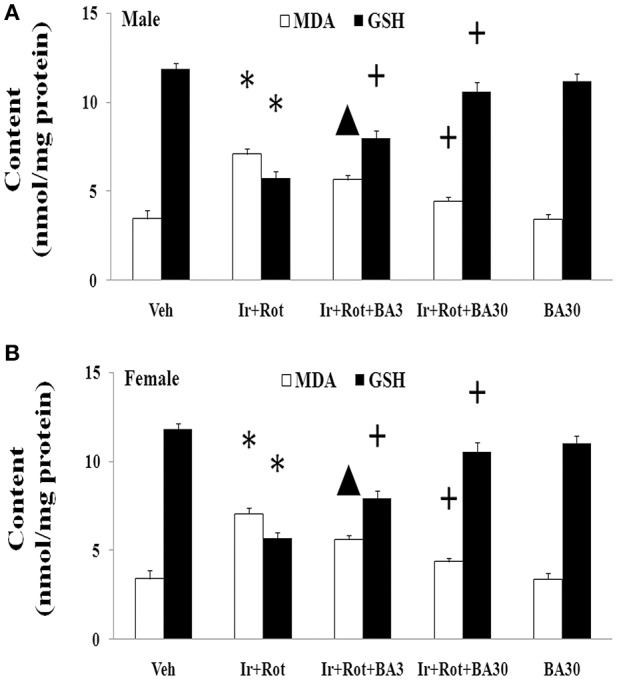
Effect of biochanin A on substantia nigra malondialdehyde and glutathione conent in male **(A)** and female **(B)** rats with iron and rotenone co-treatment. Results are expressed as mean ± SEM. *N* = 9. MDA and GSH content were analyzed using multi-factor ANOVA followed by Bonferroni *post hoc* test. ^*^p < 0.01, compared with the vehicle-treated rats. ^▲^*p* < 0.05, compared with the rats co-treated with iron and rotenone. +*p* < 0.01, compared with the rats co-treated with iron and rotenone. MDA, malondialdehyde; GSH, glutathione; Veh, vehicle; Ir, iron; Rot, rotenone; BA3, biochanin A (3 mg/kg); BA30, biochanin A (30 mg/kg).

Finally, we investigated the potential mechanism underlying biochanin A's neuroprotection in the male and female rats with iron and rotenone co-treatment. As shown in Figure 9, treatment with biochanin A significantly decreased malondialdehyde [*p* < 0.05 (3 mg/kg), *p* < 0.01 (30 mg/kg)] content and increased glutathione (*p* < 0.01) content in the substantia nigra of male and female rats co-treated with iron and rotenone compared with the Ir+Rot group.

## Discussion

Up to now, it remains unclear about the etiology and pathogenesis of PD. The “multiple hit” hypothesis that multiple risk factors may act together to induce PD neurodegeneration is a widely-accepted potential mechanism for PD (Sulzer, 2007; Ghanbari et al., 2016; Kim, 2017). Researchers estimated ambient exposures to the pesticides in California's heavily agricultural central valley, showing patients exposed to paraquat, ziram, and maneb together experienced increased risk for PD, suggesting that pesticides, which are key environmental factors, may act together to induce dopaminergic neurodegeneration (Wang A. et al., 2011). Nowadays, studying the combined effect of several risk factors is being become a hot topic in PD research.

In our present study, the rats with increased neonatal iron (120 μg/g bodyweight) supplementation were treated with a relatively low dose of rotenone (0.5 mg/kg) for 35 days when they were aged to 14 weeks. We observed that although no significant behavioral and neurochemical change was observed in the male and female rats treated with iron (or rotenone) alone compared with the vehicle-treated rats, iron and rotenone co-treatment significantly induced behavioral and neurochemical deficits in the male and female rats on the 45th day after the last rotenone injection. Our results suggest that increased neonatal iron supplement may enhance susceptibility of dopaminergic neurons to subsequent exposure of environmental toxins and increase risk for the development of PD, even though environmental toxins may be at a relatively low dose which is not harmful for ordinary individuals in our daily life. In addition, no significant behavioral change was observed in the male and female rats co-treated with iron and rotenone on the 15th day after the last rotenone injection until on the 45th day, suggesting that the effect of iron and rotenone co-treatment on behavior indexes was in a time-dependent manner in the male and female rats. Clinical and epidemiologic studies show that there are distinctions in incidence and disease progression of PD between men and women (Baldereschi et al., 2000; Taylor et al., 2007; Alves et al., 2009; Pavon et al., 2010). The data that men are more likely to develop PD and more severe phenotype than women highlight the importance of dissecting gender disparities in PD research. Therefore, based on previous studies (Dluzen et al., 1996; Chen H. et al., 2015; Wu et al., 2016), male and female rats were respectively, compared in our study. In the present study, no significant difference was observed in the sensitivity to iron and rotenone co-treatment between male and female rats. The difference of our results from others' may be attributable to different experimental samples and conditions. Some gender differences in epidemiologic and clinical features of PD may be attributable to many factors including gender-related biological and environmental differences. Although iron is important for human body especially for neural development, disruption of iron metabolism may be involved in the etiopathogenesis of PD, which is indicated by the evidence that iron levels are selectively increased in the substantia nigra of PD patients and pharmacological or genetic chelation of iron could exert neuroprotective effect in animal models of PD (Sofic et al., 1991; Griffiths et al., 1999; Kaur et al., 2003; Gaeta and Hider, 2005; Stayte and Vissel, 2014; Ward et al., 2014). In addition, direct injection of ferric iron into the substantia nigra was observed to lead to behavioral changes associated with dopamine depletion in the striatum (Lin and Ho, 2000; Junxia et al., 2003). Peripheral iron overload resulted in loss of dopaminergic neurons in rats (Jiang et al., 2007). Moreover, the correlation between increased dietary iron supplementation and PD has also been reported in aging rats (Kaur et al., 2007; Chen H. et al., 2015). At present, infant iron-fortified formula is increasingly popular as an alternative to breastfeeding for iron supplementation (Hare et al., 2015). Therefore, the possible neurotoxicity of increased dietary iron supplementation should be seriously taken into consideration. Further studies are needed to determine optimal dose of iron for infancy dietary intake.

Oxidative stress is believed to play a crucial role in the pathogenesis and development of PD (Jensen and Oliveira, 2014; Niranjan, 2014; Javed et al., 2016; Jiang et al., 2016; Niedzielska et al., 2016; Xie and Chen, 2016; Tio et al., 2017; Zhao et al., 2017). Oxidative stress is the result of an imbalance between pro-oxidant and antioxidant causing the generation of toxic reactive oxygen species (Barnham et al., 2004; Metherell et al., 2016; Hefti et al., 2017). In recent years, multiple studies have suggested that iron, as well as rotenone, potentially results in the formation of reactive oxygen species (ROS) such as superoxide free radical and may consequently contribute to inducing selective and progressive dopaminergic neurotoxicity (Gao et al., 2002; Sherer et al., 2003a; Kaur et al., 2007; Sanders and Greenamyre, 2013; Zhang et al., 2014). In the present study, co-treatment with iron and rotenone significantly induced malondialdehyde (a product of oxidative damage to lipids) increase and glutathione (an antioxidant) decrease in the substantia nigra of male and female rats, indicating that iron and rotenone co-treatment may act together to induce behavioral and neurochemical deficits through inducing redox imbalance. Moreover, there was no significant change in cerebellar malondialdehyde and glutathione content of the rats co-treated with iron and rotenone, suggesting regional selectivity of redox imbalance induced by co-treatment with iron and rotenone. Of the various populations of neurons in the brain, substantia nigra dopaminergic neurons are particularly vulnerable to oxidative insult. Enhanced susceptibility of dopaminergic neurons to oxidative damage so far has been mainly attributed to their decreased intrinsic antioxidant capacity (Jenner and Olanow, 1998; Gao et al., 2002; Wang X. J. et al., 2011). In the present study, iron and rotenone co-treatment significantly decreased substantia nigra TH expression while no significant change was observed in the substantia nigra TH expression of male rats treated with iron (or rotenone) alone. Therefore, we infer that elevated neonatal iron supplement may produce moderate oxidative stress, which does not induce significant dopaminergic neurotoxicity. It is triggered by a second hit such as rotenone in later life, and then exaggerated oxidative stress may seriously impair dopaminergic neurons. Collectively, redox imbalance may be a mechanism for dopaminergic neurotoxicity induced by iron and rotenone co-treatment. Further studies will be needed to investigate precise mechanism underlying dopaminergic neurotoxicity induced by iron and rotenone co-treatment.

Biochanin A, an O-methylated isoflavone, is extracted from soy, chickpea or red clover. Being a natural compound, biochanin A is traditionally used as a carminative, antispasmodic, expectorant, and emmenagogue in some countries (Wang et al., 2014). Moreover, biochanin A may improve lipid profile as a peroxisome proliferator-activated receptor gamma (PPARγ) agonist and provide beneficial effects on metabolic syndrome such as hyperglycaemia (Wang et al., 2014; Park et al., 2016). It has been reported that biochanin A may be useful in the prevention and treatment of breast cancer, prostate cancer and hepatocellular carcinoma (Sun et al., 1998; Khan et al., 2011; Chen J. et al., 2015; Youssef et al., 2016). In addition, biochanin A was shown to exert neuroprotective effect in neurodegenerative diseases via inhibiting microglia activation or attenuating L-glutamate cytotoxicity (Chen et al., 2007; Tan et al., 2013; Wang J. et al., 2016). Here, we showed that biochanin A could improve behavioral and neurochemical deficits in male and female rats with iron and rotenone co-treatment in a dose-dependent manner. Malondialdehyde content decrease and glutathione content increase were also observed in the substantia nigra of male and female rats co-treated with iron and rotenone after the administration of biochanin A, indicating that maintaining redox balance may be a potential mechanism for neuroprotection by biochanin A.

In conclusion, our results show that co-treatment with iron and rotenone may result in aggravated neurochemical and behavioral deficits in male and female rats through inducing redox imbalance. Increased neonatal iron supplementation may enhance susceptibility of dopaminergic neurons to subsequent exposure of rotenone. Biochanin A may exert dopaminergic neuroprotection by maintaining redox balance.

## Author contributions

XW conceived the research and designed the experiments. XW, LY, HC, ZY, MW, and YL performed the experiments and collected the results. XW, LY, and HC statistically analyzed the data. XW, LY, and HC interpreted the results. The manuscript was written and revised by XW and LY. All the authors critically reviewed the manuscript and approved the final submitted manuscript.

### Conflict of interest statement

The authors declare that the research was conducted in the absence of any commercial or financial relationships that could be construed as a potential conflict of interest.
